# Fine-Scale Analysis of Runs of Homozygosity Islands Affecting Fertility in Mares

**DOI:** 10.3389/fvets.2022.754028

**Published:** 2022-02-17

**Authors:** Nora Laseca, Antonio Molina, Manuel Ramón, Mercedes Valera, Florencia Azcona, Ana Encina, Sebastián Demyda-Peyrás

**Affiliations:** ^1^Laboratorio de Diagnóstico Genético Veterinario, Departamento de Genética, Universidad de Córdoba, Córdoba, Spain; ^2^Cersyra de Valdepeñas, Instituto Regional de Investigación y Desarrollo Agroalimentario y Forestal Castilla La Mancha, Tomelloso, Spain; ^3^Departamento de Agronomía, Escuela Técnica Superior de Ingeniería Agronómica, Universidad de Sevilla, Sevilla, Spain; ^4^IGEVET (UNLP-CONICET LA PLATA), Facultad de Ciencias Veterinarias, Universidad Nacional de La Plata, La Plata, Argentina; ^5^Asociación Nacional de Criadores de Caballos de Pura Raza Española, Sevilla, Spain; ^6^Departamento de Producción Animal, Facultad de Ciencias Veterinarias, Universidad Nacional de La Plata, Buenos Aires, Argentina; ^7^Consejo Nacional de Investigaciones Científicas y Técnicas (CONICET LA PLATA), La Plata, Argentina

**Keywords:** runs of homozygosity, genomics, reproductive efficiency, mares, inbreeding, SNP, fertility, ROHi

## Abstract

The loss of genetic variability in livestock populations bred under strict selection processes is a growing concern, as it may lead to increased inbreeding values and lower fertility, as a consequence of the “inbreeding depression” effect. This is particularly important in horses, where inbreeding levels tend to rise as individuals become more and more closely related. In this study, we evaluated the effect of increased inbreeding levels on mare fertility by combining an SNP-based genomic approach using runs of homozygosity and the estimation of genetic breeding values for reproductive traits in a large population of Pura Raza Española mares. Our results showed a negative correlation between whole-genome homozygosity and fertility estimated breeding values (EBVs) at the genome level (*ρ* = −0.144). However, the analysis at chromosome level revealed a wide variability, with some chromosomes showing higher correlations than others. Interestingly, the correlation was stronger (−0.241) when we repeated the analysis in a reduced dataset including the 10% most and least fertile individuals, where the latter showed an increase in average inbreeding values (F_ROH_) of around 30%. We also found 41 genomic regions (ROHi, runs of homozygosity islands) where homozygosity increased 100-fold, 13 of which were significantly associated with fertility after cross-validation. These regions encompassed 17 candidate genes previously related to oocyte and embryo development in several species. Overall, we demonstrated the relationship between increased homozygosis at the genomic level and fertility in mares. Our findings may help to deal with the occurrence of inbreeding depression, as well as further our understanding of the mechanisms underlying fertility in mares.

## Introduction

The increase in average inbreeding rates has become a serious concern in wild and livestock populations over the past two decades (1, 2). This phenomenon leads to a decrease in phenotypic values in fitness and fertility traits (3) known as inbreeding depression. At the molecular level, inbreeding depression is triggered by differences in the homozygosity load in regions associated with the genetic architecture of the traits. For this reason, two individuals with the same inbreeding value (estimated from pedigree and/or molecular methodologies) may show increased homozygosity in different regions of the genome, thus affecting its phenotype to a different extent (4).

In horses, increased inbreeding is of particular concern. In closed studbooks, such as Pura Raza Española (PRE) (5) and Thoroughbreds (6), the ban on using non-registered individuals as breeders tends to drastically reduce the effective population sizes and increases the number of closely-related matings which are the main cause of this phenomenon. However, breeds with open studbooks, such as Polo ponies, have also been affected, probably by increased selection intensity in mares, driven by the use of large-scale embryo transfer programs and cloning (7, 8).

Reproductive traits are extremely sensitive to problems derived from high levels of inbreeding (9). This can be even more problematic in horses due to their moderate reproductive ability, probably due to matings being mostly chosen for a combination of sports performance, morphological traits and pedigree lineages, without taking into account fertility as a selection criterion (10). In addition, fertility in mares is difficult to evaluate on a population basis, since large, reliable phenotypic datasets are needed to analyze quantitative traits with low heritabilities, and these are extremely scarce (11). However, some phenotypic traits which can be estimated directly from pedigree records have recently been validated in large-scale studies assessing mare fertility (10, 12), providing an interesting method of obtaining highly accurate reproductive estimated breeding values (EBVs). Among these, a mare's reproductive efficiency (Re), estimated as the percent deviation from the optimal number of foals produced at the end of her reproductive life, has recently been proposed as a reliable alternative in terms of heritability and accuracy in the PRE breed (13), enabling us to determine estimated breeding values (EBV's) in large datasets of mares.

Nowadays, horse breeding is entering the genomic era at full gallop. The number of individuals genotyped has increased exponentially over recent years, and it is now common to find studies including genotypes of hundreds of individuals (14–17). For this reason, the approach to determining the potential causes of inbreeding depression is shifting from the analysis of pedigree records to the use of genomic estimations based on runs of homozygosity (ROH) (18). This Methodology was originally developed in humans (19) and is currently the state-of-the-art technique for analyzing the effects of inbreeding on livestock populations (20). In addition, ROH analysis allows us to associate increased homozygosity in a specific genomic region with a phenotypic effect in large populations (21). Using this approach, Nani and Peñagaricano (22) have recently determined the existence of specific genomic regions where increased homozygosity negatively affected fertility in Holstein bulls. Likewise, Metzger et al. (23) used a similar methodology to evaluate the effect of inbreeding in reproductive traits in a small population of horses. However, to our knowledge, this approach has not yet been used to evaluate the relationship between homozygosity and reproductive traits in large populations of mares.

In this study, we used an ROH-based methodology to analyze the relationship between homozygosity and fertility in a large cohort of PRE mares. In addition, we performed a complete genome scan to determine the existence, topological position, and putative function of genomic regions where increased homozygosity could negatively affect fertility.

## Materials and Methods

### Reproductive Phenotypes

The initial reproductive dataset included 344,707 foaling records from 78,986 breeding mares belonging to 8,133 studs included in the PRE studbook. Records were obtained from the breed's creation until 2020, and only those of mares born after 1970 were kept, when the breed's official parentage control was established. Mares whose main activity was not foal production (at least 10 foals per year) were excluded, as were mares mainly devoted to leisure or sports activities (first foaling after 7 years old, with an interval between first and second foaling, and last and penultimate foaling over the age of 5).

Individual horse fertility was determined by assessing the reproductive efficiency (Re), which was defined as the percentage relationship between the current and optimal parity number during a mare's entire reproductive life, or the last known age of mares which were still reproductively active. This reproductive trait was recently validated by our group as an indirect estimator of the mare fertility in a large population of PRE horses (13).

### Genetic Evaluation and Re_EBV_ Deregression

Measurements of Re_EBV_ were estimated using a restricted maximum likelihood (REML) animal model as follows:


y=μ+age+F+Xb+Zu+e;

where **yi** is the vector of the dependent variable (Re), **μ** is the overall mean, **b** is a vector of fixed effects, including *stud, size of the herd in which the mare was born* and *year of birth*; **age** and **F**, are the age and inbreeding of the mare respectively, included as lineal covariates, **u** is the vector of random effects due to the additive animal genetic effect, and **e** is the vector of the residual error, while X and Z are incidence matrices that relate the fixed and random effects with the dependent variable.

The expected variances of the model are:


$$Var\left[\begin{array}{c} u\\ e \end{array}\right]={\left[\begin{array}{cc} A\sigma_{a}^{2} & 0\\ 0 & I\sigma_{e}^{2} \end{array}\right]}_{\;:\;E(u)=0\;and\;E(e)=0}$$

The pedigree data of mares and all known relatives (*n* = 87,227) included in the additive genetic relationship matrix (A) (24) averaged 5.65 and 9.46 complete and equivalent generations available, respectively, with a maximum of 16. All the data was provided by the Asociación Nacional de Criadores de Caballos de Pura Raza Española.

All the calculations were performed using the RENUMF90, PreGSF90, and AIREMLF90 modules from the BLUPF90 software family (25). As a final step, Re_EBV_ were deregressed to pseudophenotypes (Re_dEBV_), following the methodology proposed by Garrick et al. (26) using the DEPROOFSF90 module from the BLUPF90 software.

### Molecular Analysis

The second step was to select 862 living individuals from 373 PRE herds for single nucleotide polymorphism (SNP) genotyping using diverse criteria, including sample availability, low average relatedness among individuals, and reliable Re_dEBV_ estimations (with minimum 85% accuracy).

### SNP Genotyping

Blood from all the individuals selected for molecular analysis was collected by jugular venipuncture using sterile EDTA tubes. DNA was obtained using the Canvax blood DNA extraction kit (Canvax Biotech, Spain) in 200 μl of whole blood according to the manufacturer's protocol. Genotyping was performed using the HD Axiom™ Equine SNP Genotyping Array (Thermofisher, Madrid, Spain), following the manufacturer's recommendations, in a Genetitan™ platform (National Genotyping Center (CeGen), Santiago de Compostela, Spain). The array included variant calls for 670,776 SNPs located uniformly across the entire genome (27). The genotypes were called using the Axiom Analysis Suite 5.0 software (Thermofisher, Spain) following the “*best genotyping practices”* workflow with default parameters (dish quality control [DQC] ≥ 0.82; call rate[CR] ≥ 97). Only SNP markers showing a high-quality genotyping rate (SNP CR > 95%) and quality (Fisher's Linear Discriminant (FLD) > 3.6) were kept, according to the manufacturer's recommendations (28). We did not estimate minor allele frequency (MAF) or perform linkage disequilibrium (LD) filtering, following the latest ROH estimation guidelines (29). The final genomic dataset included 540,294 SNPs per individual, located in 32 chromosomes.

### Individual Inbreeding Coefficients Based on ROH Estimation

Whole-genome homozygosity characterization was performed by analyzing the ROH load in the R statistical environment V4.1 (30). We first determined the number, position and length of ROHs per animal using the *slidingRuns* procedure in the DetectRUNS Package (31), with the following parameters: *windowSize* = 50, *minSNP* = 100, *threshold* = 0.05, *maxGap* = 100,000, *minLengthBps* = 1,000,000, *maxOppWindow* = 1, *maxMissWindow* = 1 and *SNPinRuns* = TRUE. These values were selected to minimize genotyping errors and to avoid detecting ROH identity-by-descent segments, following Meyermans et al. (29). Finally, the molecular inbreeding (F_ROH_) (percentage of the genome covered by ROH) per individual was estimated at genome and chromosome levels, following McQuillan et al. (19).

### Relationship Between Whole-Genome Homozygosity and Fertility

To test the relationship between whole-genome homozygosity and Re, we first estimated the non-linear correlations between F_ROH_ (whole and per chromosome) and Re_dEBV_ in the 862 individuals genotyped. Next, we selected a reduced dataset (RD) which included individuals showing the top (High-Fert) and bottom (Low-Fert) 10% Re pseudophenotypes (*n* = 172), to increase the power of the association test. In both cases, the estimations were performed using the *nlcor* package (32) from R. Finally, we compared F_ROH_ values (at the genome level and per chromosome) between High-Fert and Low-Fert individuals to find differences in homozygosity associated with variations in fertility traits.

### Detection of Genomic Regions Significantly Associated With Fertility

The genomic regions of the RD in which the incidence of ROH increased significantly (ROH islands, ROHi) were determined using a permutation test (1,000,000 iterations per marker) as described by Goszczynski et al. (4). Only regions larger than 100,000 bp which showed a 100-fold increase [-log_10_(*p*-value) > 2] compared with a random occurrence probability were tagged as ROHi and further analyzed.

### Validation of Significant ROHi in the Whole Population

The ROHi identified in the RD were validated in the whole dataset using the method described by Nani and Peñagaricano (22). Briefly, the association between each ROH region and the Re values was determined using a *t*-test; ROHs with a *t*-value ≥ 2 were confirmed as significantly associated with mare fertility.

### Analysis of Significant Regions and Candidate Genes

The regions showing a significant association were transformed into *GRanges* objects and intersected with the most recent version of the horse genome [EquCab 3.0, (33)], using the *HelloRanges* package in R (34), retrieving all the genes located within the candidate regions. Thereafter, the functionality of each gene was annotated using DAVID (35) and PANTHER (36) bioinformatic resources. Finally, we performed a comprehensive literature review using public scientific databases such as PubMed and Scopus to outline the possible association between candidate genes and fertility.

## Results

### Genetic Characterization of Reproductive Traits

**Figure 1:**
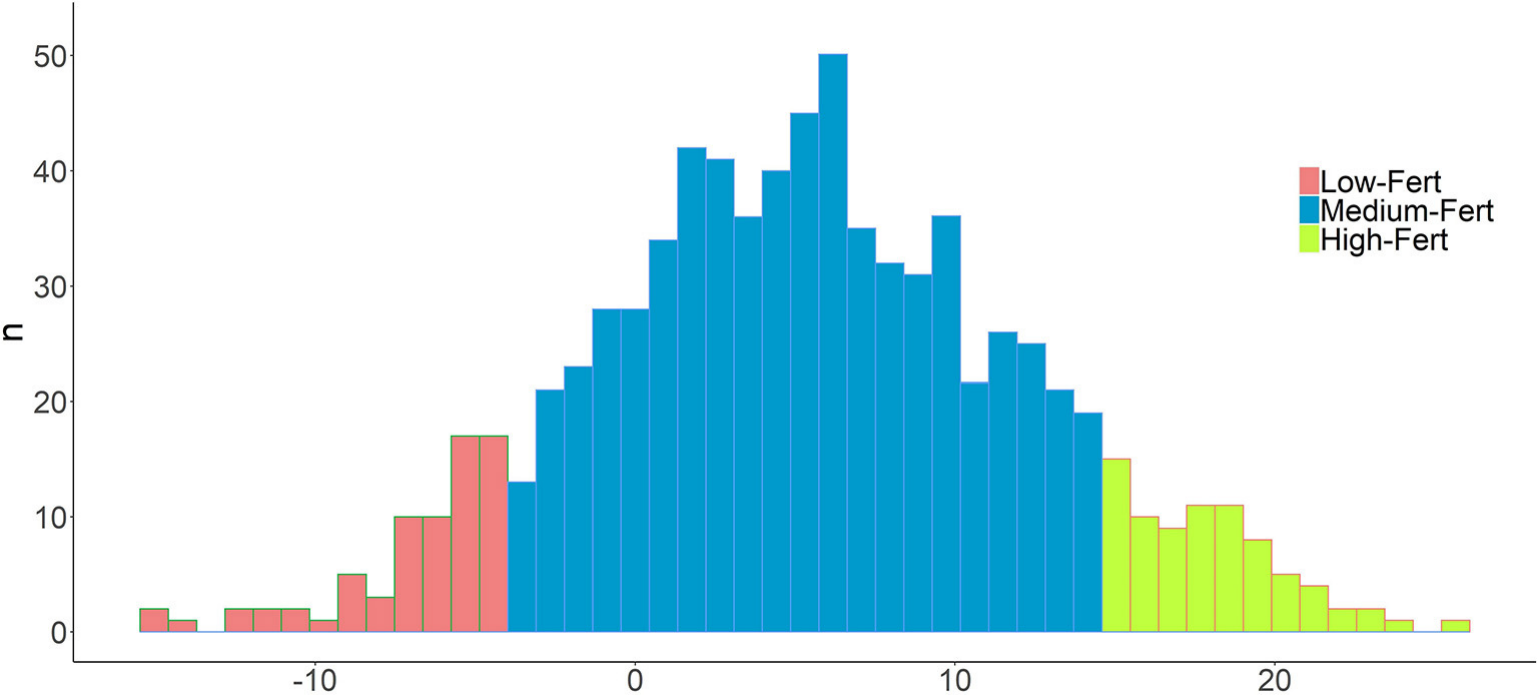
Distribution of Reproductive efficacy pseudophenotypes (Re_dEBV_) in the whole population. Values of the top and bottom 10% individuals are shown in green and red, respectively. A normal distribution is followed (Shapiro test).

Re_EBV_ heritability (h^2^) was 0.25 ± 0.0031, revealing a substantial genetic effect. In addition, the standard deviation was nearly 80 times lower, which demonstrates their reliability. In addition, the Re_EBV′s_ of the individuals genotyped showed a normal distribution (Shapiro-Wilk test, *P* > 0.05), while displaying a wide range of variability (−15.16 to 25.52; Figure 1). Finally, the average values of the Low-Fert (*n* = 82; −15.16 to −3.60) and High-Fert (*n* = 82; 14.63 to 25.62) clusters were highly divergent, which allowed us to perform a more robust analysis in terms of fertility (Figure 1).

### Estimations of Molecular Homozygosity (ROH Analysis)

**Figure 2:**
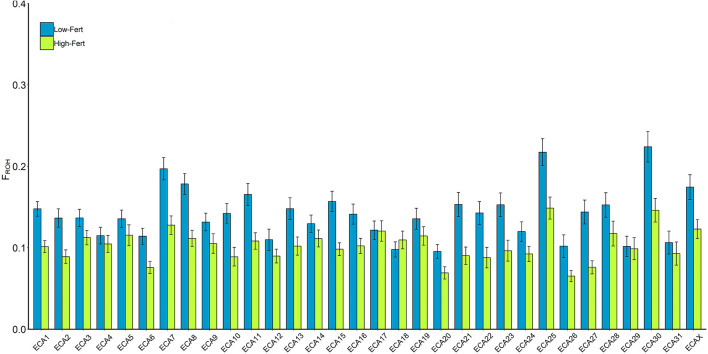
F_ROH_ per chromosome in low (blue) and high (green) fertility groups. The values were estimated as the percentage of each chromosome covered per ROH. ^*^Indicates significant (*p* < 0.05) differences within chromosomes between groups.

The average F_ROH_ in the whole population was high (0.136 ± 0.07). However, it was nearly 30% lower in High-Fert individuals (F_ROH_ = 0.102 ± 0.06) compared with Low-Fert (F_ROH_ = 0.141 ± 0.07). Similar results were obtained in the analysis per chromosome, in which High-Fert individuals showed a lower F_ROH_ than Low-Fert in 19 chromosomes (*t*-test; *P* < 0.05) (Figure 2; Supplementary Table 1).

### Correlation Between Genomic Homozygosity and Re

**Figure 3:**
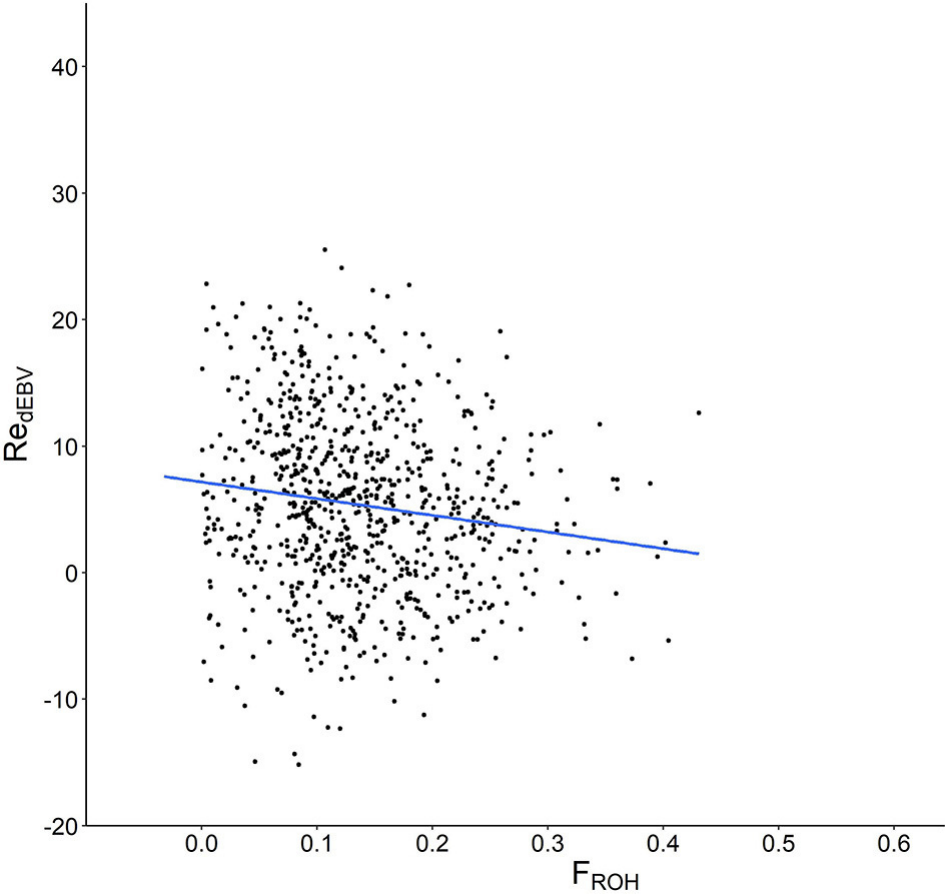
Scatter plot showing the relationship between molecular homozygosity (F_ROH_) in the x axis and the estimated breeding values for mare fertility (Re_dEBV_) in the y axis.

The correlation between Re and the whole genomic homozygosity estimate (FROH) was negative and highly significant (ρ = −0.147, *P* < 0.0001, Figure 3). As expected, this value was even more negative in the RD (ρ = −0.241; *P* < 0.01). However, differences were not evenly detected across the different chromosomes (Supplementary Table 2). For example, the correlations per chromosome estimated in the whole population were negative but highly variable (ranging from −0.033 in ECA30 to −0.134 in ECA15), whereas correlations in the RD showed a similar pattern, but were higher than those observed in the previous analysis. Interestingly, the correlations between Re and FROH were only significant in 18 and 15 of the 32 chromosomes evaluated in the whole population and RD, respectively, suggesting the existence of genomic regions more or less involved in the genetic control of fertility.

### Detection of ROHi and Candidate Genes Associated With Fertility

**Figure 4:**
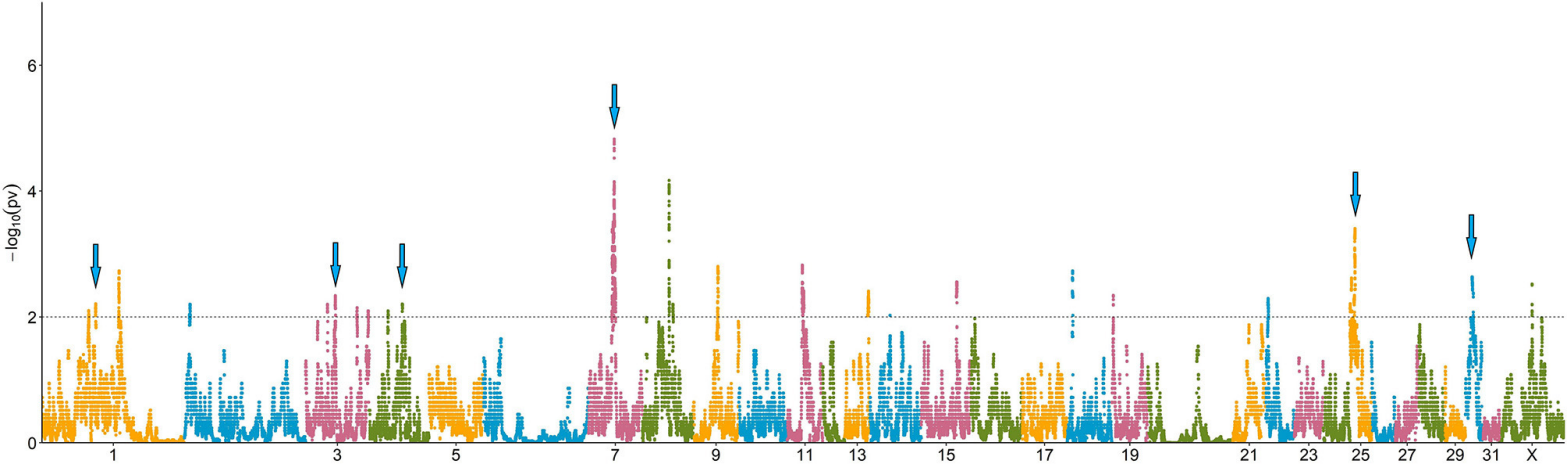
Manhattan plot depicting the incidence of ROH per marker (estimated as -log_10_(*p*-value) using a permutation test). Significance was set at a 100-fold increase (dotted line). Cyan arrows show ROHi statistically associated with Re after validation.

In total, 41 genomic regions (covering 39.04 Mb in 17 chromosomes) showed a 100-fold increase in ROH abundance in the RD (*P* < 0.01; Figure 4; Supplementary Table 2). Only 13 of these regions (located on six chromosomes) were significantly associated with Re_dEBV_ after validation in the whole population (*n* = 862; *t-test, P* < 0.01, Table 1). Of these, three different regions—located in ECA3, 7, and 25—included 17 genes previously associated with biological processes related to female fertility, including oocyte and embryo development.

**Table 1:** ROH islands significantly associated with Re_dEBV_ validated in the whole population (*n* = 862).

**Region**	**Chromosome**	**Start**	**End**	**Size**	***p*-value**	***T*-value**	**Genes related to fertility**
ROH2	1	97,899,186	98,904,589	1,005,403	0.009339	0.022255	
ROH7	3	40,269,277	41,449,687	1,180,410	0.008175	0.000125	*ADH5, EIF4E*
ROH12	4	62,610,358	63,234,819	6,24,461	0.00727	0.046533	
ROH14	7	44,206,164	49,253,548	5,047,384	0.000016	0.009749	*ASF1B,DHPS, DNAJB1, DNMT1, GIPC1*,
							*KEAP1, NDUBFB7, PRDX2, PRKACA, PTGER1*,
							*RFX1, S1PR2, SPC24, and TYK2*
ROH15	7	49,5154,21	49632,127	1,167,06	0.000092	0.00432	
ROH16	7	49,891,501	54,438,682	4,547,181	0.008277	0.01323	
ROH33	25	4,756,726	4,864,928	108,202	0.008348	0.011066	
ROH34	25	8,239,464	9,343,625	1,104,161	0.009448	0.000259	
ROH35	25	9,919,290	10,142,948	223,658	0.000554	0.030493	
ROH36	25	10,231,120	11,142,083	910,963	0.003169	0.001147	*SMC2*
ROH37	30	11,750,277	12,881,858	1,131,581	0.003673	0.001564	
ROH38	30	14,317,398	14,418,301	100,903	0.004307	0.016978	
ROH39	30	15,102,347	15,212,221	109,874	0.009435	0.016161	

*Start, End and Size, expressed in bp. P values, were obtained using a permutation test, following Goszczynski et al. (4). T-values were obtained using a t-test following Nani and Peñagaricano (22)*.

## Discussion

In this study, we measured the potential effect of increased homozygosity on fertility in mares using a combined approach of high-throughput genomic analysis and genetic estimations of fertility. We explored further into which putative mechanisms, biological processes and genes might be involved in this phenotypic effect by analyzing two groups of individuals with divergent fertility EBVs. Although the negative relationship between inbreeding and fertility has already been demonstrated (37–40), this is the first analysis to use a genomic approach in mares. In addition, our analysis was performed in a breed with a high rate of inbred individuals (41), as well as a wide genetic variability for fertility in mares (10), making it a very interesting experimental model.

### Relationship Between Homozygosity and Fertility at Genome and Chromosome Levels

Our results demonstrated a strong, negative correlation between genomic homozygosity (F_ROH_) and fertility (Re_dEBV_), in line with previous reports in this species (39, 40). However, this is the first time this correlation has been obtained by combing genetic estimations and an SNP-based molecular approach. In addition, the analysis of the RD showed a ≈ 40% decrease in homozygosity rates in High-Fert mares, suggesting that the relationship between fertility and inbreeding depression in mares may be based on a threshold model (42), which could explain why the effect is more noticeable when inbreeding values rise above certain values.

Interestingly, the correlations between fertility and homozygosity at chromosome level were highly variable in both whole and reduced datasets. Chromosomal variability in terms of ROH has been recently reported in several horse breeds, such as Mangalarga (43), Halflinger (44), and Norwegian–Swedish (45), although it was not related to changes in fertility in these cases. However, a similar variable pattern in the association between homozygosity load at chromosome level and fertility was reported by Martikainen et al. (46) in Finnish cows. Since reproductive traits are polygenic, some genomic regions obviously explain better the changes observed in breeding values than others. This fact is also supported in our study by the fact that the chromosomes showing the strongest (ECA15) and weakest (ECA18 and ECA29) correlations remained unchanged, regardless of the dataset analyzed. However, the lack of correlation observed in some chromosomes also coincides with the hypothesis proposed by Howard et al. (47), who demonstrated that this null effect could be explained by the co-existence of ROH and homozygous variants in the same region, with unfavorable and favorable outcomes, thus producing a neutral effect. In addition, this variable pattern can also explain the variability in the inbreeding depression load recently reported by our group (41), where some highly inbred individuals showed an increased incidence of morphological defects, whereas others with similar inbreeding values did not. Finally, it is important to mention that the highest correlations between fertility and genomic homozygosity were obtained in the analysis of whole-genome data rather than per chromosome. This fact suggests we should be cautious when using chromosome-level homozygosity values as predictors of inbreeding depression in polygenic traits (or at least in fertility), since genes from several chromosomes probably contribute to the inbreeding depression in fertility and should therefore be taken into account globally.

### Effect of Increased Homozygosity on Fertility at Genome Level

Pryce et al. (48) demonstrated that inbreeding depression can be reduced in cattle by avoiding the increased homozygosity in small, specific areas of the genome. These regions, currently known as ROHi, are nowadays the state-of-the-art methodology for studying the mechanisms affected by inbreeding in a given trait (20). However, they can only be reliably measured by using fine-scale genomic methodology and analyzing large populations. In horses, this approach has been used recently to measure regions associated with inbreeding depression in morphological traits in breeds bred for different purposes (21, 43), as well as to evaluate the effect of inbreeding on fertility in stallions (23). Thus far, this methodological approach has not been used in the analysis of fertility in mares.

Our experimental design included the analysis of 172 individuals, showing the highest and lowest Re_dEBV_ values from a large population of 850 animals. This methodology, which includes divergent phenotypes, is commonly used in GWAS studies to increase detection power (49) and to determine the effect of inbreeding on fertility in bulls (22); however, it had not been previously used to evaluate the effect of inbreeding depression on female fertility in livestock species. Using this methodology, we detected 41 regions in which the homozygosity rate (at SNP level) had increased significantly (over 100 times greater than the rate of chance occurrence); in addition, 13 of them were associated with changes in fertility, after validation in the whole population (850 horses). This finding concurs with Pryce et al. (48), who demonstrated that increased homozygosity in certain regions can explain the occurrence of inbreeding depression in a specific trait better than others. However, it also highlights the fact that, in order to obtain more reliable results, the analysis of inbreeding depression should be performed in small genomic regions rather than at chromosome or whole genome levels. In addition, the fact that only 13 of 41 regions detected were associated with variation in the fertility of the mare is in agreement with Nani and Peñagaricano (22), who demonstrated that performing a cross-validation test of each specific ROHi against the genetic (or phenotypic) value in a large population is a vital step to avoid the detection of false-positive associations between inbreeding and phenotypes.

### Functional Analysis of Candidate Regions

Three genomic regions associated with changes in fertility (ROH7, 14, and 36) contained 17 genes previously related to female reproductive competence in several species. None of them had been previously mapped as QTLs or harbored mutations and/or indels associated with fertility in horses (11). Interestingly, these three regions were among the four regions most closely associated with R_edEBV_. However, there were no genes associated with fertility in the 10 remaining regions, which showed an association with R_edEBV_w of a lower magnitude.

The genomic region showing the most significant association with R_edEBV_ was located on ECA3, including two major genes related to female fertility: *alcohol dehydrogenase 5* (*ADH5*) and *eukaryotic translation initiation factor 4E* (*EIF4E*). In mice, inhibition of the *ADH* family reduced the de-novo production of retinol acid in the ovaries and the response to treatment with equine chorionic gonadotropin (50), resulting in decreased developmental competence and quality of the oocytes ovulated. Similarly, *eIF4E*, known as cap-binding protein, has been associated with the onset of translational activity and meiotic maturation in mammalian oocytes of several species by repressing early oogenesis (51–53). However, when it becomes phosphorylated, the limitation is released (54). This metabolic step has been described as crucial for efficiently initiating the translation and activation of the MAP kinase pathway in the oocyte, which increases the synthesis of several proteins related to spindle formation, allowing the acceleration of oocyte maturation after progesterone induction (52).

ECA25, another region associated with fertility, includes the *structural maintenance of chromosomes 2* (*SMC2*) gene. Its main function is to encode one of the subunits which form the canonical condensin complex (*condensin I*) in mitotic and meiotic cells (55). *Condensin I* is required for many functions of meiotic chromosome dynamics (chromosome individualization, resolution, and segregation) and therefore plays a key role in oogenesis progress and blastocyst formation (56).

However, it was a 5Mb genomic region located in ECA 7 which showed the greatest abundance of genes related to female reproduction, although they all showed more indirect associations with fertility, rather than a direct and causative association. Among these, *PTGER1* (*Subtype 1 Prostaglandin E Receptor 1*) is related to ovarian regulation and the onset of decidualization during the pre and post-implantation periods in mice (57); *ASF1B* modulates and stabilizes chromatin remodeling during the replication-dependent assembly (58), *DNAJB1* and *NDUFB7* are related to oocyte aging and insulin-dependent changes in mitochondrial activity, respectively, in cattle (59, 60); and *GIPC1* has been identified as a part of the *IGF1* receptor on *Xenopus spp*. during oocyte metabolism (61). Similarly, the expression of *PRDX2* in external vesicles has been suggested as a predictor of embryo implantation ability (62), while *RFX1* and *PRKA2* were related to embryonic development failure and lethality in mice (63, 64). The fact that none of these genes were mentioned as pivotal infertility processes suggests that they may be involved in the modulation of fertility in mares by a polygenic pathway, via small genetic effects (11). Despite the fact that this hypothesis should be treated with caution since it was not validated in our study, it supports the idea that using individuals with a reduced inbreeding load (13) for fertility traits may prove an interesting option for mitigating the effect of inbreeding on mare fertility.

In conclusion, we demonstrated for the first time the existence of a negative relationship between increased homozygosity at the genomic level and fertility in a large population of mares. In addition, we proved the existence of small genomic regions in which increased homozygosity negatively affects their fertility to a greater extent. Finally, our functional analysis showed that several genes related to fertility in other species were located within the genomic regions, showing the strongest association with fertility. Our findings can be therefore considered a first step toward determining putative genomic regions which account for the relationship between inbreeding and fertility in mares, which in turn may help to limit the effect of inbreeding depression on fertility in mares.

## Data Availability Statement

The original contributions presented in the study are included in the article/Supplementary Material, further inquiries can be directed to the corresponding author/s.

## Ethics Statement

Ethical review and approval was not required for the animal study. DNA samples, and genealogical and reproductive data were provided by the ANCCE studbook.

## Author Contributions

SD-P, AM, MR, and MV: project conceptualization and experimental design. MR, AE, SD-P, and AM: methodology. AE, MV, and NL: data gathering. NL, FA, MR, and SD-P: data Analysis. NL, AM, MR, and SD-P: manuscript write and review. MV, AM, and SD-P: funding acquisition. AM and SD-P: project management. SD-P: project coordination. All authors contributed to the article and approved the submitted version.

## Funding

Funds for this project were obtained from an AGL2017-84217-P grant (MINECO, Spain, PI: AM) and a PICT-2018-0227 grant (ANPCyT, Argentina, PI: SD-P).

## Conflict of Interest

The authors declare that the research was conducted in the absence of any commercial or financial relationships that could be construed as a potential conflict of interest.

## Publisher's Note

All claims expressed in this article are solely those of the authors and do not necessarily represent those of their affiliated organizations, or those of the publisher, the editors and the reviewers. Any product that may be evaluated in this article, or claim that may be made by its manufacturer, is not guaranteed or endorsed by the publisher.
