# Supplementary material for: Fine-Scale Analysis of Runs of Homozygosity Islands Affecting Fertility in Mares
Source: Front Vet Sci. 2022 Feb 17;9:754028. doi: 10.3389/fvets.2022.754028 (PMC8891756; doi:10.3389/fvets.2022.754028)
Supplement: Supplementary file 1 [file Table_1.docx]

Supplementary Material

## Supplementary table 1

F_ROH_ values per chromosome and group. P-values in bold were significant (p<0.05, T test)

Supplementary Table 2

Pearson correlations between F_ROH_ and Re_dEBV_ in the whole population and the reduced dataset. P-values in bold were significant (p<0.05, T test)

Supplementary Table 3


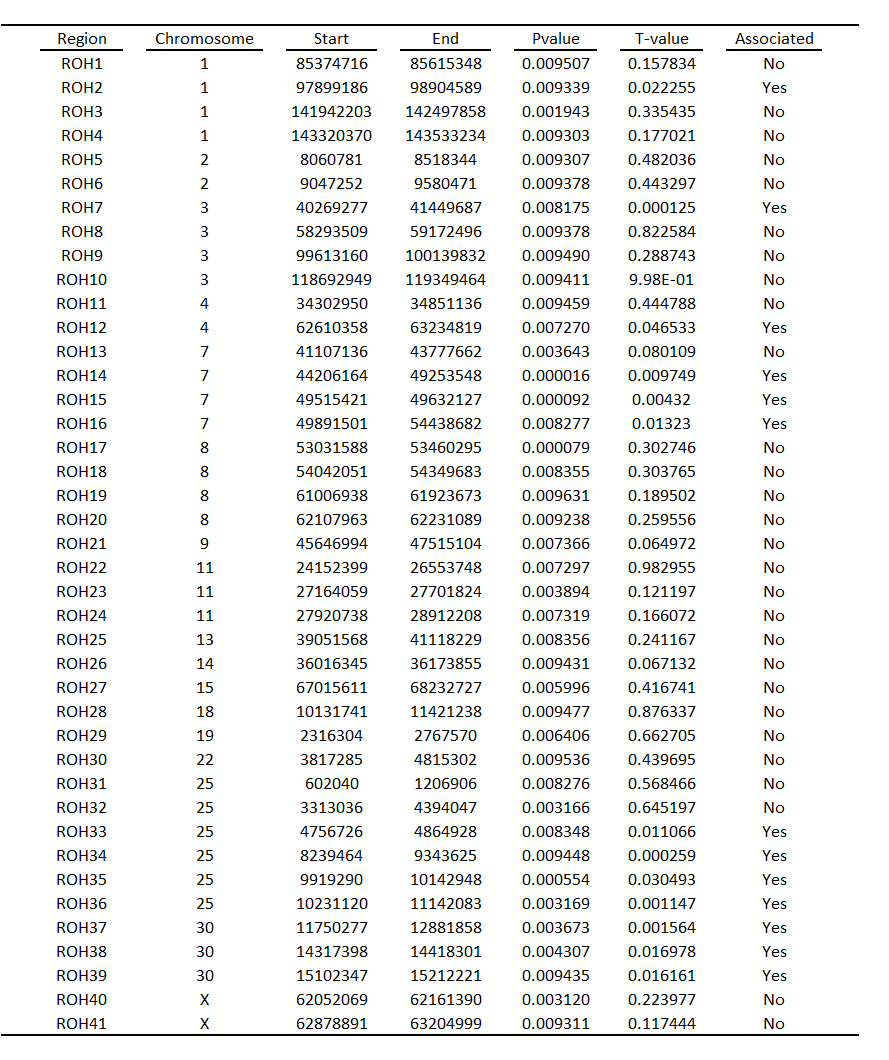
Genomic regions showing a 100X increase in the homozygosity. Start, end and size are expressed in bp. P-values were obtained using a permutation test according to Goszczynski et al. (2018). T-values were obtained using a T-test according to Nani and Peñagaricano (2020).
